# Learning while evaluating: the use of an electronic evaluation portfolio in a geriatric medicine clerkship

**DOI:** 10.1186/1472-6920-6-4

**Published:** 2006-01-12

**Authors:** Gustavo Duque, Adam Finkelstein, Ayanna Roberts, Diana Tabatabai, Susan L Gold, Laura R Winer, members of the Division of Geriatric Medicine, McGill University

**Affiliations:** 1 Division of Geriatric Medicine, McGill University, Montreal, Quebec, Canada; 2 Centre for Medical Education, McGill University, Montreal, Quebec, Canada; 3 Instructional Multimedia Services, McGill University, Montreal, Quebec, Canada; 4 Office of the Deputy Provost and CIO, McGill University, Montreal, Quebec, Canada

## Abstract

**Background:**

Electronic evaluation portfolios may play a role in learning and evaluation in clinical settings and may complement other traditional evaluation methods (bedside evaluations, written exams and tutor-led evaluations).

**Methods:**

133 third-year medical students used the McGill Electronic Evaluation Portfolio (MEEP) during their one-month clerkship rotation in Geriatric Medicine between September 2002 and September 2003. Students were divided into two groups, one who received an introductory hands-on session about the electronic evaluation portfolio and one who did not. Students' marks in their portfolios were compared between both groups. Additionally, students self-evaluated their performance and received feedback using the electronic portfolio during their mandatory clerkship rotation. Students were surveyed immediately after the rotation and at the end of the clerkship year. Tutors' opinions about this method were surveyed once. Finally, the number of evaluations/month was quantified. In all surveys, Likert scales were used and were analyzed using Chi-square tests and t-tests to assess significant differences in the responses from surveyed subjects.

**Results:**

The introductory session had a significant effect on students' portfolio marks as well as on their comfort using the system. Both tutors and students reported positive notions about the method. Remarkably, an average (± SD) of 520 (± 70) evaluations/month was recorded with 30 (± 5) evaluations per student/month.

**Conclusion:**

The MEEP showed a significant and positive effect on both students' self-evaluations and tutors' evaluations involving an important amount of self-reflection and feedback which may complement the more traditional evaluation methods.

## Background

Recently, the evaluation of medical students in clinical settings has changed from the traditional apprenticeship model with written examinations and tutor-led evaluations to a more structured set of methods that includes modular evaluations, log books and portfolios [1]. This change in the educational environment has forced the evolution of new kinds of evaluation instruments moving from a predominantly summative to a more formative evaluation which benefits student learning in several ways.

In the case of portfolios, they allow for authentic formative evaluation by providing students with the opportunity to learn while being evaluated [1,2]. In fact, portfolios allow for the evaluation to be structured and criterion-based [3]. Students are evaluated not only at the end of each rotation, they are provided with feedback throughout the rotation leading to incremental improvements during the apprenticeship and the opportunity to share and improve their performance. Furthermore, portfolios allow for a more complete picture of student learning in which students can track the history of their improvements over time and reflect upon their actions at various time points during the apprenticeship [4].

Traditional evaluation methods are mostly tutor-led and include written exams, tutor-ratings reports and bedside assessments. These methods are known to be based on patient availability and are not always explicitly aligned with any predefined learning objectives [5]. By contrast, evaluation portfolios encourage reflective practice [4,5]which provides students with the opportunity to reflect upon the skills that they have been working on throughout the rotation, examine their progress and implement action plans for improvement thus encouraging self-regulated learning. However, some limitations to the effective use of evaluation portfolios have been reported [1]. Amongst these limitations, the novelty of the method is the factor that has significantly affected student utilization of this evaluation systems, additionally, tutor participation has been limited for still unidentified reasons.

The implementation of McGill University's clerkship in Geriatric Medicine [6] included a new method of evaluation to stimulate self-reflection in clerks as well as student-tutor interaction. This method is the McGill Electronic Evaluation Portfolio (MEEP), a web-based electronic portfolio designed to facilitate the use of portfolios in clinical settings making it accessible from any computer with an Internet connection. This paper presents the results of the evaluation of the MEEP. In conducting our evaluation, we hypothesized, first, that students may require an effective hands-on introduction into the use of portfolios in order to improve their performance in the MEEP as a new evaluation and learning method. Second, we also hypothesized that the MEEP as a web-based system may facilitate the process of evaluation in clinical settings where time constraints pose a significant limitation, improving student and tutor perceptions of the evaluation process and finally increasing the number of tutor-student interactions.

## Methods

The MEEP is a web-based portfolio that has been integrated into the clerkship using WebCT, Inc.'s course management system (WebCT) as its main platform. The characteristics of and theoretical basis for the MEEP have been previously outlined [4]. Briefly, the MEEP includes a list of ten skills and eight attitudes in which the students are expected to demonstrate the acquisition of competence during a four-week rotation. Students may post their self-reflections and action plans every time they perform one of the skills or are exposed to an attitude-related situation while dealing with geriatric patients. Working from input from the students and feedback from their tutors and members of the multidisciplinary team (nurses, social workers, physiotherapists, etc.), the system tracks the skills and attitudes that they have completed, are working on, and have not yet performed to give them real-time feedback on their progress. Tutors post feedback and comments to their assigned students' portfolios as well as observe and contribute to the feedback and comments given to students they are not assigned to evaluate, but with whom they may interact. The system builds a learning curve for each specific skill and attitude, based on both student and tutor observations [7].

The portfolio represented 20% of their final grade. They were expected to complete satisfactorily at least eight out of ten skills. If students do not complete more than eight skills satisfactorily, students receive 2 points (out of 20) per completed skill. By contrast, the attitudes only had a qualitative value in the global evaluation of the student. For 80% of the final grade, students were evaluated based on traditional tutor-led lecture and teaching sessions, specifically using the evaluation report known as the Practice of Medicine (POM) evaluation form, a rating form used in most of the clinical clerkships at McGill University whose categories run from Unsatisfactory to Superior [8].

The study participants were McGill University 3^rd ^year medical students completing their mandatory four-week clerkship rotation in Geriatric Medicine. On the first day of each rotation, students in one group attended an introductory session given by the clerkship coordinator. This session took place in a computer laboratory equipped with personal stations allowing for hands-on practice with the portfolio and the web-based system. To test if this introductory session had an effect on the students' ability to work with the portfolio, the introductory session was not given to 50% of the rotation periods, which were randomly selected. For those students who did not receive an introductory session, access to an explanatory website with all the relevant information was offered [9]. Furthermore, a thirty-question mandatory electronic survey was completed by the students at the end of their rotation which included the global assessment of their experience in Geriatric Medicine. Among those thirty questions, three questions were dedicated to soliciting their opinions about the MEEP. Additionally, a second optional survey was sent to the same set of students at the end of the clerkship year. This second survey consisted of thirty-six questions, including the same three questions asked on the first survey concerning the MEEP. In the first two questions, a five-point Likert scale ranging from "strongly disagree" to "strongly agree" was used. By contrast, for the third question students were asked to rate the portfolio as a complement to the traditional evaluation method (written exams, tutors' rating reports and bedside evaluations) on a five-point scale ranging from poor to excellent.

Additionally, all tutors involved in student supervision and evaluation were surveyed at the end of the academic year. The ten questions comprising this electronic survey included not only tutor considerations about the usefulness of the MEEP, but also potential limitations to making better use of the portfolios and giving feedback more effectively. A five-category Likert scale ranging from "strongly disagree" to "strongly agree" was used. Finally, the number of postings by both students and tutors were also tracked and registered. The data were analyzed using SPSS v12.0. Chi-square tests and t-tests were performed to assess significant differences in the responses from surveyed subjects. All the results are reported using mean ± SD.

## Results

**Figure 1:**
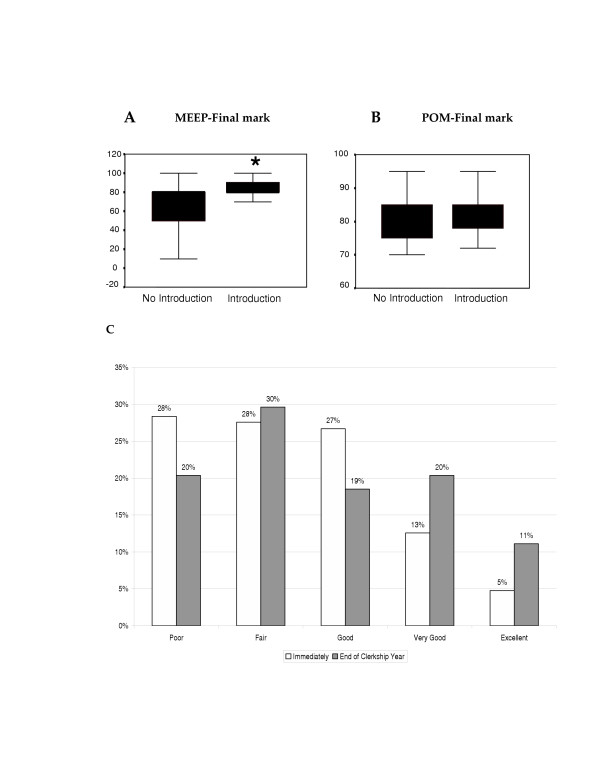
**A and B. Effect of the introductory session on students' performance in their MEEP and POM evaluations**. The figure shows the average scores of MEEP (panel A) and POM form (panel B) for the 77 students who received an introductory session vs. 47 who did not receive it. A significant difference was found between both groups in the MEEP while no difference for POM marks was found (*p < 0.001). **C. Student ratings of the electronic portfolio as an evaluation tool**. Using a 5-point Likert scale students were asked to rate the MEEP as an evaluation tool using a scale ranging from Poor to Excellent both immediately and post-clerkship. The figure shows a significant positive change in the number of students who considered the MEEP as very good or good evaluation tool immediately after their rotation versus at the post-clerkship survey (p < 0.04).

A total of 133 third-year medical students (11 students per month) were evaluated using the MEEP between September 2002 and September 2003. For the 77 students who received an introductory session their average MEEP score at the end of their rotation was 8 out of 10 skills (80%) satisfactorily completed; by contrast, for those students who did not receive an introductory session, the average was 66%. A significant difference was found between both groups (p < 0.001). Interestingly, when the same analysis was pursued for the more traditional POM rating form no significant difference between both groups was found (Figure 1, A and 1B).

Complete survey data was available for 130 of the 133 students who both used the MEEP during the clerkship and submitted the first mandatory survey at the end of their rotations. Seventy-two students (55 %) completed and submitted the second voluntary survey at the end of the year. To assess the user-friendliness of this method, students were asked to rate how comfortable they felt using the MEEP. The initial survey immediately after the rotation showed that of students attending introductory sessions, 66% "strongly" or "somewhat agree" to feeling comfortable using the MEEP, while among those who did not attend intro sessions, only 48% reported "strong" to "somewhat" agreement that they were comfortable with the MEEP (p < 0.05). In the second survey, a significant positive change (57%) was found in the number of students who "strongly" agreed (p < 0.04) (Table 1) as well as a significant reduction in the number of students who disagreed (data not shown). No differences were found between those students who received the introductory session and those who did not. Additionally, when students were asked if the electronic portfolio brought more effective and continuous feedback to the traditional evaluation system, a significant positive change in the number of students who "somewhat" or "strongly" agreed was seen between the first and the second surveys (21% vs. 26%) (p < 0.04) (Table 1). In this case, the introductory session did not have any affect on students responses. Finally, when students were asked to rate the portfolio as an evaluation method in clinical settings in addition to traditional evaluation methods, there was a positive change in the number of students who considered it "very good" or "excellent" rising from 18% to 31% (p < 0.04) (Figure 1C).

Furthermore, a total of eighteen out of thirty (60%) tutors completed the tutor survey. Fifty percent agreed that the criteria for evaluating each category were clear (Table 2). In addition, 80% of the tutors felt comfortable using the electronic portfolio (Table 2). Finally, when tutors were asked to weight the different factors that act as limitations to their use of the electronic portfolio, 70% considered time to be a strong or moderate limitation followed by the clarity of the evaluation criteria (60%). In contrast, only 20% and 30% of the tutors respectively considered computer literacy and the absence of a helpdesk to be a limitation (Table 2).

**Figure 2:**
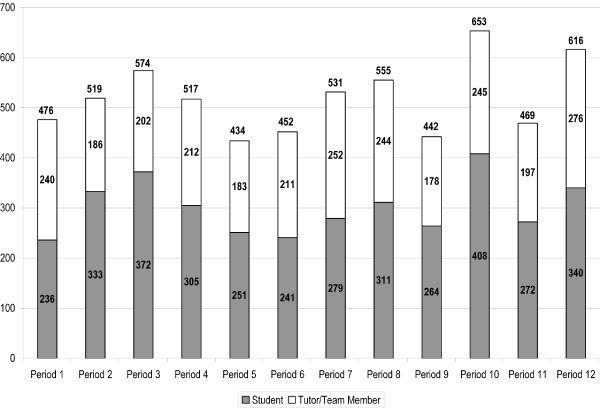
**Number of evaluating postings per month by students and tutors**. Throughout the academic year 2003–2004 we had an average of 520 ± 70 postings/month with a tutor/student ratio of 1.3 indicating a slightly higher proportion of students' postings.

Finally, we looked at the number of postings per rotation by both students and tutors. As shown in Figure 2, throughout the 2002–2003 academic year we had an average of 520 (± 70) postings/month with 30 ± 5 evaluations per student in a month. Only those evaluations that included comments and action plans, and therefore self-reflections, were registered and counted.

## Discussion

Portfolios allow for authentic formative, structured and criterion-based evaluation and provide the opportunity for self-reflection by students [10]. The first reports of the use of student portfolios in clinical settings to evaluate student acquisition of clinical skills came from nursing schools, which demonstrated their usefulness as an efficient evaluation tool and learning method [11]. By contrast, medical schools have only recently implemented their own portfolios at both the undergraduate and postgraduate levels with differing results [12-14]. In both undergraduate and postgraduate levels, the use of written portfolios has similar limitations including the time constraints of clinical practice, the lack of familiarity with the method and the additional amount of paperwork required to complete them [3]. These limitations have resulted in negative feedback from students and tutors about the use of portfolios in clinical settings [13].

The MEEP was designed to be powerful, yet simple and convenient to use; its web-based nature means that users can create an evaluation quickly at any time and from any computer. Within these evaluations, students and tutors are expected to write comments and action plans that stimulate further self-reflection and on-site learning thus transforming the e-portfolio from a static repository into a dynamic cycle of evaluation fed by student-tutor interaction thus increasing the immediacy of the feedback [4].

To address our first hypothesis we found that student performance in the portfolio was worse and the experience was considered less comfortable when they did not receive the introductory session. The difference in student performance in the portfolio after the implementation of the introductory session may be explained by the fact that despite the information available on our website, students reacted better to using the MEEP because they received a more dynamic explanation of it and were able to ask questions thus better understanding its value to the skill development process. As expected, this introductory session did not have an effect on students performance in their tutor-led evaluations (POM forms).

Our second goal was to identify the effect of the MEEP on the learning environment by examining its user-friendliness and acceptance as a new evaluation method. In some cases, Geriatric Medicine was the students' first clinical rotation leaving some students with no benchmark for comparing their experience with other types of evaluation when answering the final survey. To reduce the effect of that handicap, we administered a second evaluative survey to identify any change in opinion about the portfolio after one year of clerkship rotations. In this study, there is a positive change over time in student conceptions of the MEEP as an evaluation method with more students considering the MEEP to be very good or excellent. Additionally, there was also a significant positive change in the number of students who feel comfortable using the MEEP. Finally, there was a slight change in student perceptions of this evaluation portfolio as a tool for more effective feedback when compared with the traditional evaluation system. However, we do not consider these results to be surprising with respect to the MEEP's acceptance because these results correlate with student perceptions of other traditional methods of evaluation such as multiple choice questions [15]. Furthermore, the student evaluations of the portfolios were not affected by the order of the clerkship rotations being similar in earlier or later clerks throughout the academic year.

Additionally, we surveyed the tutors in an attempt to determine their acceptance to this method and the potential limitations to their use of the portfolio. In general, the method was well accepted by the tutors. Indeed, we found that time (or lack thereof) was the main limitation to the tutors' ability to use the electronic portfolio and to provide more frequent feedback. This means that while the MEEP does not solve all time constraints, tutor limitations to the MEEP do not relate largely to the general system of evaluation.

Finally, we would like to underscore the significant change in the learning environment represented by the quantity of the student and tutor postings. We think that this is the most important strength of this method since very few evaluation methods provide such a number of evaluation events (thirty evaluative postings with feedback) in a short period of time (one month). Since only students evaluations that included comments and action plans were accepted as valid, we found that every posted evaluation represented an active exercise of self-reflection that is usually wanting in other evaluation methods. A good example of the quality of the self-reflection process is shown in our sample portfolio [7].

A potential limitation to our study is that the improvement in the MEEP's acceptance using student survey data after one year may be due to a regression phenomenon. However, we consider that limitation to be mitigated by including the set of students who completed geriatrics as the last rotation of the academic year and who had already experienced all other traditional and new evaluation methods in their previous clerkship rotations throughout the year.

A second limitation is the fact that tutors postings do not necessarily follow the students self-assessment limiting the effectiveness of the tool as a negotiated-learning tool. However, we found in both feedback surveys, from tutors and students, that they recognize the complementarity of this tool to the usual daily interactions between tutors and students. In fact, in some cases tutors sat down with their students to fill out their MEEP evaluations and to go over their list of skills.

In summary, an analysis of our findings shows that although an introductory session is required, the MEEP is a new evaluation method that encourages student self-reflection, tracks student progress in skill acquisition and stimulates student-tutor interaction with a high level of acceptance by tutors and students. Moreover, this tool may represent a valuable complement to the most traditional evaluation systems in clinical settings.

## Competing interests

The author(s) declare that they have no competing interests.

## Authors' contributions

GD conceived of the study, participated in the conception and design of the portfolio and helped draft the manuscript. AF participated in the design of the portfolio and helped draft the manuscript. AR participated in the design and coordination of the study, performed statistical analysis in the paper and helped draft the manuscript. DT performed statistical analysis in the paper. SG helped conceive of the design of the study and helped revise the manuscript. LW helped conceive of the design of the study and helped revise the manuscript. All authors read and approved the final manuscript.

## Pre-publication history

The pre-publication history for this paper can be accessed here:



## Figures and Tables

**Table 1:** Comparison of surveyed students' evaluations of the portfolio immediately after their rotation vs. at the end of their clerkship years. Data shown for students who strongly or somewhat agree.

		**Immediately**	**End of clerkship year**	**P**
I felt comfortable using the MEEP	With introductory session	66 %	57 %	< 0.04
	Without introductory session	48 %	56%	< 0.04
MEEP helps as a tool for more effective and constant feedback	With introductory session	21 %	26 %	< 0.04
	Without introductory session	19 %	27 %	<0.04

**Table 2:** Tutors' opinions regarding the use of the McGill Electronic Evaluation during a clerkship rotation in Geriatric Medicine (n = 18).

	**Strongly/somewhat agree**	**Neither agree or disagree**	**Strongly/somewhat disagree**	**NA**
The criteria for evaluating each category are clear.	50 %	20 %	10%	20%
I feel comfortable using the electronic portfolio.	80%	0%	20%	0%

**Table 3:** Tutor limitations to using the MEEP (n = 18).

**Rate the degree to which the following factors act as limitations to your using the electronic portfolio.**				
	**Strong Limitation**	**Moderate Limitation**	**No Limitation**	**NA**
Time	40%	30%	20%	10%
Electronic Portfolio Training	0%	30%	60%	10%
Computer Literacy	10%	10%	70%	10%
Helpdesk availability	10%	20%	40%	30%
Evaluation Criteria	30%	30%	20%	20%
